# Drivers of avoidable emergency department visits in older adults with multiple long-term conditions and palliative care needs: a qualitative survey across six care providers

**DOI:** 10.1080/02813432.2026.2709432

**Published:** 2026-08-12

**Authors:** Hanna Bring, Monica Bergqvist, Karin Modig, Pia Bastholm-Rahmner, Katharina Schmidt-Mende

**Affiliations:** a Karolinska Institutet, Department of Neurobiology, Care Sciences and Society, Division of Family Medicine and Primary Care, Stockholm, Sweden; b Academic Primary Health Care Center, Stockholm, Sweden; c Karolinska Institutet, Department of Neurobiology, Care Sciences and Society, Division of Nursing, Stockholm, Sweden; d Karolinska Institutet, Institute of Environmental Medicine, Unit of Epidemiology, Stockholm, Sweden

**Keywords:** Emergency department visits, older adults, palliative care, primary health care, multiple long-term conditions

## Abstract

**Objectives:**

Emergency department (ED) visits remain common among older adults with multiple long-term conditions, many of whom have palliative care needs. However, the ED environment is burdensome for these patients and often misaligned with their goals. This study explores experiences of shortcomings in outpatient care that drive ED use in this population to inform a primary care intervention.

**Method:**

Qualitative content analysis of free-text responses from a survey of 100 physicians, registered nurses and nurse assistants working across primary care practices, home healthcare, EDs, geriatrics, ambulance services and advanced home healthcare in Stockholm.

**Results:**

The analysis distinguished one overarching theme: ‘Delayed, fragmented palliative care in primary care settings contributes to ED visits’. This theme comprised two categories: (1) Palliative decision-making in primary care, including subcategories *Recognizing palliative care needs and initiating care*, and *Including patients and families in shared decision-making*; and (2) Delivery of palliative care at home, including sub-categories *Care planning and symptom management at home*, and *Ensuring information transfer regarding patients’ preferences*. Respondents described how these shortcomings led to crisis-driven ED visits that often conflicted with patients’ wishes to remain and die at home.

**Conclusion:**

Delayed recognition of palliative care needs and the resulting delay in action are perceived as important drivers of avoidable ED visits in older adults with multiple long-term conditions. Strengthening generalist palliative care in primary care through targeted education, adequate resources, and access to specialist support are potential important components of an intervention aiming to reduce ED use and enable more proactive, person-centred, end‑of‑life care.

## Introduction

The number of older adults living with multiple long-term conditions is steadily increasing [1,2]. As these conditions progress, frailty – characterized by declining functions and decreased resilience to stressors – becomes more common and pronounced [3]. Older adults with multiple long-term conditions and frailty often experience a high symptom burden and complex healthcare needs and may therefore benefit from a palliative care approach [4–6]. Palliative care aims to improve quality of life for individuals with life-threatening illness by managing symptoms and providing care aligned with patients’ values and preferences [7]. Importantly, the need for palliative care may arise long before the final phase of life and can be addressed alongside curative or life-prolonging treatments [8].

Many older adults with palliative care needs prefer to remain at home for their care [9,10], yet many die in hospital, often against their stated wishes [9,11]. Emergency department (ED) visits often increase in the end of life [12–14], which is potentially concerning as the ED can be a challenging environment for older adults with palliative care needs, potentially exposing them to burdensome investigations when they are assessed by healthcare professionals unfamiliar with their medical history [13,15]. While palliative care does not eliminate the need for emergency care, its early integration can reduce crisis-driven ED visits by addressing unmet needs such as pain or shortness of breath [12,14].

Previous studies, mostly conducted outside Sweden, have identified low access to palliative care and inadequate symptom management as a key driver of ED visits at the end of life [16,17]. The underlying reasons for insufficient symptom control are likely complex and interrelated, including healthcare system conditions such as limited access to primary care and social support, as observed in the broader population of older adults [18,19]. However, it remains unclear if these aspects are equally relevant among older adults with multiple long-term conditions and palliative care needs. Furthermore, most research has focused on patients already enrolled in specialised palliative care – predominantly those with cancer. However, the generally proposed model for palliative care is the generalist-specialist approach: healthcare professionals in primary care and nursing homes provide generalist palliative care, while specialist palliative care serves as the primary provider for complex cases [8,20,21]. Less is known about older adults with multiple long-term conditions and palliative care needs who are not receiving specialized services, many of whom have symptoms related to non-cancer conditions [16,20].

Understanding healthcare professionals’ experiences on why older adults with palliative care needs visit the ED is essential for optimizing home-based care and supporting end-of-life preferences. Overall, little is known about how unmet palliative care needs affect older adults with multiple long-term conditions and contribute to avoidable ED visits in outpatient care settings. Moreover, previous research has largely examined individual healthcare providers such as outpatient palliative care, primary care or EDs. Context-specific information is therefore needed to inform future intervention development [22].

To address this gap, we draw on data from a survey on 100 healthcare professionals, originally designed to describe perceived causes of avoidable ED visits among older adults with multiple long-term conditions. The survey design allowed for the collection of perspectives from professionals across different care settings, providing a broad view of outpatient care delivery. In the primary study (manuscript submitted), respondents identified limited access to care, insufficient healthcare professional competence, communication deficiencies, and unclear professional responsibilities as important contributors to ED visits.

During qualitative analysis, we interpreted issues related to palliative care needs as a prominent and analytically distinct aspect that extended beyond the scope of the primary study, indicating a need for a focused exploration. Therefore, the aim of this study was to identify experiences of shortcomings of outpatient care contributing to avoidable ED visits among older adults with multiple long-term conditions and unmet palliative care needs. The findings are intended to inform the development of a complex intervention in primary care.

## Material and methods

### Study design

This study is based on analysis of a dataset of open-ended survey responses originally collected in a study on avoidable ED visits among older adults with multiple long-term conditions. During the initial analysis, material relating to palliative care in primary care settings (Box 1) was interpreted as a distinct analytic focus and subsequently re-examined in a separate analysis. Given the importance of these findings for the care of older adults with palliative care needs, we considered it important to explore and present this material in sufficient depth rather than treating it as a minor component of the primary analysis.

Box 1. Description of healthcare context

In this study, primary care settings refer to continuous, comprehensive, first-contact healthcare providers, including primary care practices, home healthcare, and nursing homes, consistent with the WHO definition of primary care [7].

Within primary care settings, informants were recruited from two healthcare provider types:

- Primary care practices, which in Region Stockholm provide routine primary care services and basic home healthcare (including wound care and medication administration) during office hours.
- Home healthcare out-of-hours (organized by healthcare providers other than primary care practices)

Outside primary care settings, informants were recruited from:

- Emergency departments (EDs)
- Geriatric care
- Ambulance services
- Advanced home healthcare (continuous 24-hour specialized care in patients’ homes, such as intravenous treatment)

Primary care practices, emergency departments, geriatric care, and advanced home healthcare share electronic medical records. Home healthcare out-of-hours and ambulance services use separate medical record systems.

In this study, we adopt a descriptive qualitative stance and understand the textual data as contextualised descriptions shaped by healthcare professionals’ experiences and interpretations [23]. We analysed the data using inductive qualitative content analysis according to Graneheim and Lundman, a method well suited for analysing textual data of varying depth and for identifying patterns across participants’ descriptions while remaining grounded in data [24]. Accordingly, the findings should be interpreted as contextualised descriptions of participants’ experiences that provide insight into why older adults with palliative care needs are admitted to emergency departments, rather than as explanations of underlying mechanisms or formal theory [24,25].

Data were collected using a web-based survey comprising three open-ended questions. The survey enabled the inclusion of healthcare professionals from diverse care settings who provide services to older adults with multiple long-term conditions.

### Study setting and target population

The study was conducted in urban and suburban areas of Stockholm, Sweden. The Stockholm region, the capital region, comprises approximately 2.5 million inhabitants, representing more than 20% of the national population. The target population consisted of healthcare professionals – nurse assistants, registered nurses, and physicians employed across six healthcare providers in outpatient and hospital settings (Box 1). We chose these professions as the target population as they are commonly involved in care processes around ED visits, but the survey was open to other healthcare professions. One physiotherapist also responded to the survey and was retained in the analysis, as the responses were considered relevant to the study aim and represented only a small proportion of the total sample. Respondents were recruited from both public and private healthcare organizations, all of which are publicly funded.

### Development of the survey questions

The survey was developed by a multidisciplinary panel consisting of physicians, registered nurses, a pharmacist, a pedagogue, and a behavioural scientist. Its primary objective was to explore healthcare professionals’ experiences of the underlying causes of potentially preventable ED visits among older adults with multiple long-term conditions. To ensure content validity and clarity, the survey was pretested with three healthcare professionals representing the ED, advanced home healthcare, and geriatrics. This pilot testing resulted in minor refinements in the wording of the questions.

The survey consisted of free-text responses to three open-ended questions as well as demographic data for the respondents. The three open-ended questions were:

1. Describe a patient case with an older adult with multiple long-term conditions where it would have been possible to prevent an ED visit. What should have been done differently, and how?
2. What changes need to be made to ensure that older adults with multiple long-term conditions avoid unnecessary ED visits?
3. Describe three or more things that you find difficult or challenging in working with older adults with multiple long-term conditions.

### Recruitment and data collection

A non-probability quota sampling approach was used to ensure representation of predefined subgroups of healthcare professionals [26], a strategy employed in qualitative research to capture diverse perspectives and experiences. Respondents were recruited *via* managers or other designated contact persons at each workplace. The managers and contact persons were recruited *via* authors’ professional networks. All were informed about the study and asked to invite 20 eligible healthcare professionals to complete the survey. The aim of this recruitment process was to include respondents with experience with the issue and the interest in elaborating their responses. Previous research suggest that this number is sufficient for studies with similar aims [27].

Inclusion criteria were at least six months of work experience. The survey was distributed by e-mail, with no incentives offered. A cover letter providing information on the study, confidentiality, voluntary participation and informed consent was included at the beginning of the e-mail. Ethical permission was granted by the Swedish Ethical Review Authority (Dnr 2022-02692-01).

Initial data collection took place between September 2022 – January 2023. Professionals from primary care practices were initially excluded, as they were planned for a separate study. However, preliminary analysis highlighted the importance of primary care-related issues contributing to ED visits, and primary care practice-professionals were subsequently recruited to provide a more comprehensive understanding. Recruitment of primary care practice professionals occurred between November 2023 and February 2024.

Although 20 respondents per target group initially agreed to participate, response rates varied. After five reminder e-mails to the initially eligible respondents, additional respondents were recruited through the same managers or contact person, who distributed the survey link directly. primary care practice professionals were recruited in a later phase *via* direct distribution of the survey link by contact persons, without reminders. The survey was completed by 100 healthcare professionals and the number of respondents varied across healthcare settings. For demographic characteristics of respondents, see Table 1.

**Table 1.** Characteristics of respondents in the original survey sample and respondents contributing the present analysis.*

	Total (*n* = 100)	ED (*n* = 23)	Geriatrics (*n* = 10)	Ambulance services (*n* = 15)	Advanced home healthcare (*n* = 18	Home healthcare out-of-hours (*n* = 12)	Primary care practices including home healthcare (*n* = 22)
Gender n(%)							
Female	67 (67)	13 (57)	9 (90)	5 (33)	13 (72)	9 (75)	18 (82)
Other/No answer	4 (4)	1 [4]	0 (0)	0 (0)	1 (6)	2 (17)	0 (0)
Age, mean (min-max)	44 (25–63)	41 (25–63)	43 (25–52)	40 (28–55)	47 (28–60)	47 (28–60)	48,5 (25–52)
Profession n(%)							
Registered nurse	67 (67)	16 (70)	1 (10)	14 (93)	14 (78)	6 (50)	16 (73)
Nurse assistant	12 (12)	1 (4)	2 (20)	0 (0)	1 (6)	6 (50)	2 (9)
Physician	20 (20)	6 (26)	6 (60)	1 (7)	3 (17)	0 (0)	4 (18)
Physiotherapist**	1 [1]	0 (0)	1 (10)	0 (0)	0 (0)	0 (0)	0 (0)
Work experience, years n (%)							
≤10	32 (32)	14 (61)	1 (10)	7 (47)	6 (33)	3 (25)	1 (5)
11–20	35 (35)	6 (26)	6 (60)	4 (27)	5 (28)	4 (33)	10 (45)
>20	33 (33)	3 (13)	3 (30)	4 (27)	7 (39)	5 (42)	11 (50)
Contributed to present analysis, n(%)	44 (44)	13 (57)	2 (20)	8 (53)	14 (78)	3 (25)	4 (18)

ED: Emergency department.

* The characteristics presented in this table refer to all respondents in the original survey sample (*n =* 100), not only those whose responses contributed to the present analysis. As the free-text responses were anonymised before analysis, individual respondent characteristics could not be linked to the respondents included in this study.

** The survey was target to nurse assistants, registered nurses and physicians. One physiotherapist answered the survey and was included in the analysis.

### Data analysis

The researchers analysed data from the open-ended questions using qualitative content analysis, following the approach described by Graneheim and Lundman [24]. Open-ended questions allows researchers to obtain a wide variety of responses, and content analysis enables systematic reduction and categorization of data in a flexible way [24].

The analysis was conducted in two stages and performed manually. In the first stage, we analysed data according to the aim of the first study. Responses were first analysed separately by workplace and then merged. To get a sense of the material, the researchers initially read all responses several times. They then systematically identified meaning units in respondents’ responses. When a respondent’s answer included different aspects of preventable ED visits, the content was divided into different meaning units. Next, meaning units were condensed and labelled with codes, interpreting the meaning of the text. Codes were compared for similarities, and those with similar content were grouped into categories and subcategories.

During this first stage of the analysis, we interpreted ED visits related to palliative care needs as a distinct aspect of the material that was not fully aligned with the aim of the first study and that deserved additional exploration. Of the 100 respondents, 44 mentioned aspects interpreted as relating to palliative care needs in at least one free text response. In the second stage, all meaning units, condensed meaning units and codes interpreted as related to palliative care needs were extracted to a separate dataset and re-analysed using the same analytical process. Finally, the researchers performed a latent analysis, examining the categories in a more abstract and interpretative way, to create an overarching theme. This theme was grounded in empirical data and reflects the context in which the described care processes primarily occurred, namely primary care and home-based care. The process of analysis is shown in Table 2.

**Table 2.** Example of the process of data analysis.

Meaning unit	Condensed meaning unit	Code	Subcategory	Category	Theme
Late identification of palliative needs hinders early contact with palliative services.	Late identification hinders early contact with palliative services.	Late identification of palliative needs	Recognizing palliative care needs and initiating care	Palliative decision-making in primary care	Delayed, fragmented palliative care in primary care contributes to ED visits
Proactive discussions with patients and relatives about goals and limits of acute care are lacking.	Absence of early discussions about goals and limits of care.	Absence of early goal-of-care discussions	Including patients and families in shared decision-making		
A palliative patient at home lacks prepared medications, leading to hospital admission for symptom relief.	A palliative patient at home lacks prepared medications, leading to hospital admission.	Lack of medication preparedness in palliative home care	Care planning and symptom management at home	Delivery of palliative care at home	
An older, multimorbid patient with Alzheimer’s disease, with documented treatment limitations, is transferred from the nursing home to the emergency department despite being assessed as naturally dying.	A naturally dying patient with treatment limitations is transferred to the emergency department instead of receiving palliative care in the nursing home.	Unnecessary hospital transfer at the end of life	Ensuring information transfer regarding patients’ preferences		

ED: Emergency department.

We have included shorter and longer quotes to illustrate the findings. Longer quotations describing patient cases were used to illustrate how respondents linked multiple interacting factors within complex care situations. In the quotations presented in the results, the researchers have converted shortening of text into full sentences, standardized terminology when necessary – for example, writing out ‘patient’ instead of the shortened ‘pat’.

### Rigor

The qualitative data analysis was conducted by three researchers (HB, MB and PBR). The results were reviewed by co-author KSM, who had read all survey responses, to ensure that no content was overlooked. All authors then participated in identifying patterns across categories through reciprocal reading of the responses in the survey text and the categories. Interpretations were continuously discussed within the research team. The research team included a behavioural scientist and a qualitative researcher (PBR), a registered nurse and qualitative researcher (MB), two physicians with clinical experience in primary care (KSM, HB). Together, these complementary clinical and methodological perspectives provided contextual understanding of the care of older adults while also requiring awareness of potential preconceptions. Throughout the analysis, we therefore continuously reflected on how our disciplinary and clinical backgrounds might influence data interpretation, challenged each other’s assumptions, and ensured that interpretations remained grounded in the respondents’ responses. This process enhanced reflexivity and contributed to the trustworthiness of the analysis [28].

## Results

**Figure 1.**
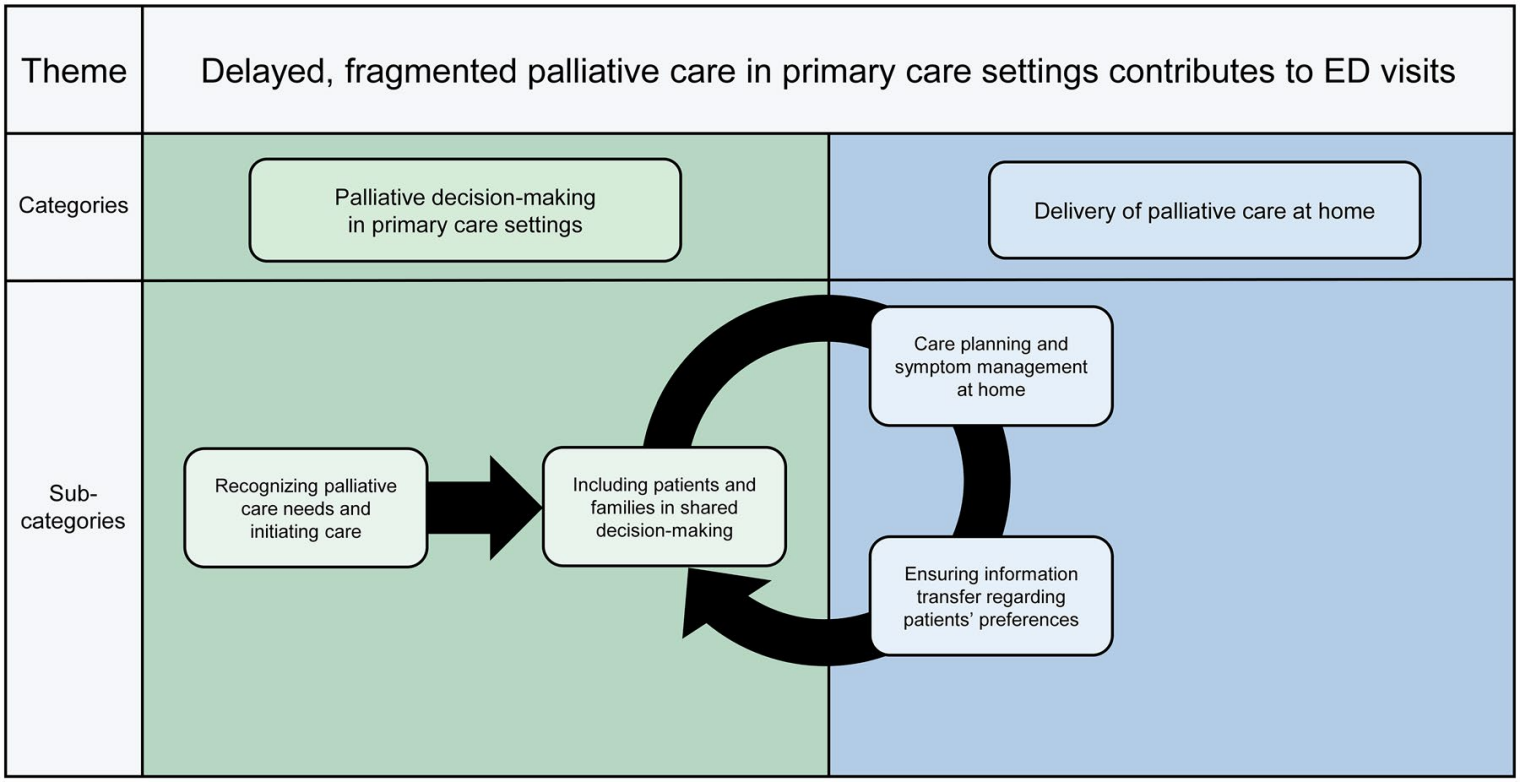
Illustration of theme, categories and subcategories from qualitative analysis. *Legend*: ED: Emergency department.

Respondents primarily described shortcomings in outpatient primary care settings (Box 1), including primary care practices, nursing homes, home healthcare both during and outside of office hours (Box 1). The analysis distinguished one overarching theme: Delayed, fragmented palliative care in primary care contributes to ED visits. The overarching theme comprises two categories, each with two subcategories: (1) Palliative decision-making in primary care, encompassing (a) Recognizing palliative care needs and initiating care and (b) Including patients and families in shared decision-making; and (2) Delivery of palliative care at home with the subcategories (a) Care planning and symptom management at home and (b) Ensuring information transfer regarding patients’ preferences (Figure 1).

### Delayed, fragmented palliative care in primary care settings contributes to ED visits

Respondents described how gaps both in initiation and delivery of palliative care in outpatient settings can contribute to ED visits in older adults with palliative care needs. Delayed identification may lead to crisis-driven decisions, while insufficient care planning and information transfer can result in care that does not align with patient preferences.

#### Palliative decision-making in primary care settings

Respondents across home healthcare, ED, geriatrics, and ambulance services described recurring challenges related to the delayed recognition of palliative care needs and the absence of early, structured discussions about treatment limitations with patients and family members in primary care settings. These interrelated issues are perceived to contribute to late and often crisis-driven decisions about end-of-life care in the ED.

##### Recognizing palliative care needs and initiating care

Respondents reported that older adults with multiple long-term conditions are at times not recognized in the early palliative phase at all, and even in a late palliative phase they may be sent to the ED without prior identification of palliative care needs. Some arrive without any prior goals-of-care discussion or established treatment limitations, forcing ED staff to make complex decisions under time pressure and with limited information. ED staff emphasized that this delayed recognition leads to unnecessary diagnostic procedures and treatments with minimal benefit, and that earlier identification could have enabled more appropriate care focusing on quality of life.

A respondent from geriatrics explicitly stated:

*Patients are identified as requiring palliative care far too late.*

Another respondent from the ED described the importance of identifying when a focus on symptom relief may be more appropriate than acute interventions:

*Is it really necessary to send a 90-year-old, frail, multimorbid patient to the emergency department? Or could greater focus be placed on symptom relief? Does the individual have sufficient reserve capacity to tolerate acute care and potentially intensive care?*

Respondents also called for updated, standardized guidelines to support timely recognition and management of palliative patients with complex needs in primary care. One respondent from ambulance services described how the lack of documented care plans and primary care support affected end-of-life care:

*This occurred outside of on-call hours. Home care services [municipal support with activities of daily life] called and reported a patient who was more or less critically ill, and according to my assessment required treatment limitations based on medical history and background. The patient absolutely did not want to go to the hospital and wished to die at home. According to the information available to us, the patient had no treatment limitations or any ongoing end-of-life care. We attempted to contact an emergency physician unit or a physician from the primary care practice for assessment and potential home-based palliative care. After multiple attempts and a long period of time, I persuaded the patient to accompany us to the ED. The patient subsequently died in the ED after treatment limitations were implemented. The patient wished to end their life at home but instead died in the ED. I received minimal support from the prehospital on-call physician, and no emergency physician or on-call physician was available, nor was timely support from the primary care possible (several hours).*

##### Including patients and families in shared decision-making

Closely related to the delayed recognition of palliative needs is the absence of early and proactive discussions with patients and families about treatment preferences and goals of care. Respondents reported that conversations regarding interventions such as cardiopulmonary resuscitation and intensive care occur too late, sometimes only after acute deterioration. This lack of preparation was perceived to cause conflicts and emotional distress among family members, who may not fully understand the meaning of the term ‘palliative phase’ or the implications of treatment limitations. One respondent from ED emphasized the importance of early discussions on goals of care:

*Early and proactive discussions with frail older adults and their relatives about goals of care […] are needed to clarify what acute hospital care can contribute to quality of life when severe illness occurs.*

Furthermore, respondents reported that these discussions should be conducted in a structured and supportive setting in primary care to promote a shared understanding and to prevent crisis-driven decision-making in the ED. A lack of mutual understanding between healthcare professionals, patients and families regarding concepts such as ‘late palliative phase’ or ‘treatment limitations’ was described as contributing to clinical decisions that did not align with the established care plan. Even when prior discussions about palliative care had taken place and the patient’s wish to remain at home was clear, anxiety and uncertainty among family members could nonetheless lead to the patient being brought to the ED. One case was described by a respondent from home healthcare:

*Received a call from the patient’s family members reporting that the patient is having breathing difficulties. I checked the patient’s medical record and saw that a geriatric physician had already conducted a treatment-limitation discussion and that the patient was classified as late palliative. The relatives requested a home visit by a physician and we planned an urgent home visit with the responsible home healthcare physician. At the same time, the relatives had already called an ambulance, and we encountered everyone there. The physician tried to explain to the family members what late palliative care means, but they had already decided that the patient should go to the ED. The physician then wrote a referral to the ED. The case was difficult to handle differently, as there was potential conflict between the primary care physician and the relatives. Perhaps the physician who conducted the treatment-limitation discussion should have explained its meaning. For me, it was difficult to determine whether the relatives had been present during that discussion or not.*

Together, these subcategories point to a systemic gap between the clinical recognition of palliative needs and the shared decision-making concerning goals of care and treatment preferences.

#### Delivery of palliative care at home

Once palliative care needs are recognized and palliative care is initiated, respondents describe gaps in the delivery of this care, especially in when generalist palliative care was provided in home-based settings. Respondents across ED, geriatric, ambulance services, home healthcare and primary care practices highlighted how fragmented communication across care providers, inadequate documentation, and lack of coordinated care planning undermine the continuity of palliative care. The importance of clear documentation and information transfer across all levels of care regarding care plans and treatment limitations was also emphasized by respondents. However, this does not always occur, resulting in care that fails to reflect patients expressed wishes and values.

#### Care planning and symptom management at home

Respondents identified deficiencies in care planning and implementation once a palliative approach had been established. When patients wish to remain at home, it is essential that a structured care plan is in place, that appropriate symptom-relief medications are readily available, and that continuous care contacts are established. Respondents described cases where these components were lacking, particularly concerning patients not enrolled in specialized palliative services such as advanced home healthcare, leaving staff without necessary medications or access to medical support. As a result, patients experiencing distressing symptoms were transferred to the ED – contrary to their preferences and at the cost of unnecessary suffering. A healthcare professional from out-of-hours home healthcare described a case:

*A patient in the end-of-life phase was sent to the ED because the home healthcare service during out-of-office hours did not have medications to relieve symptoms (e.g., pain, rattling breathing, anxiety). Such situations need to be anticipated by the primary care practice′s home healthcare service during daytime hours, i.e., by prescribing these medications in advance and keeping them accessible at the patient’s home for the on-call nurse. Sending a patient like this to the ED only causes unnecessary suffering for both the patient and their relatives.*

Limited access to acute palliative care in primary care settings such as home healthcare was reported as a factor contributing to potentially avoidable ED visits. As a solution, one respondent from geriatrics suggested establishing mobile home care teams closely integrated with specialized palliative care units.

*Connections with palliative care units could be established, allowing direct admissions. A home healthcare team could potentially be linked to the palliative care unit to facilitate continuity of care and ongoing contact within the healthcare system*

Such teams could provide support to patients with complex needs and reduce unnecessary ED visits and hospitalizations.

##### Ensuring information transfer regarding patients’ preferences

Respondents described how fragmented information flow between nursing homes, home healthcare, primary care practices, and ambulance services can lead to uncertainty about patients’ treatment goals and status. Even when patients have clearly expressed their preferences or treatment limitations, this information may not be recorded or accessible in medical records. Consequently, healthcare professionals may initiate interventions that contradict the patient’s prior decisions. One respondent from ED highlighted this issue:

*Sometimes older patients arrive where it appears that treatment limitations have not been assessed before an ambulance is called and the patient is sent to the hospital.*

In several cases, respondents reported that patients who had declined hospital-based interventions or expressed a wish to die at home were nonetheless transferred to the ED due to missing documentation or unclear communication between healthcare professionals. Respondents reported a need for better documentation systems and standardized routines for transferring and updating palliative care information to ensure that patients receive care consistent with their preferences. A professional from the ED described case of a patient referred from a nursing home.

*An older, patient with multimorbidity residing in a nursing home has, among other conditions, end-stage renal failure but has declined dialysis and prefers end-of-life care. The patient was brought to the ED in the middle of the night because, for unclear reasons, the nursing home had taken blood tests earlier in the day, showing a creatinine level above 900. The patient was woken in the middle of the night for ambulance transport to the ED. The patient was not in pain, anxious, or otherwise distressed, and had already declined dialysis. The outcome was that the patient was admitted to the hospital for palliative care. The patient had already expressed their treatment preferences and would likely have been better off remaining in their home environment with good nursing care.*

## Discussion

We found that interrelated shortcomings in outpatient settings, mainly at primary care practices, in home healthcare and at nursing homes, contributed to ED visits among older adults with multiple long-term conditions and palliative care needs. Delayed recognition of palliative care needs and initiation of palliative care, insufficient shared decision-making with patients and family members, fragmented communication between providers, poor documentation and absence of care planning all contribute to potentially avoidable ED visits. Limited access to palliative support in the home further compounds these issues. Together, these findings underscore the need to develop, and test coordinated and integrated approaches to generalist palliative care within primary care.

In this study, challenges in identifying palliative care needs in primary care were evident. While these difficulties have been described in previous research [8,29], our findings illustrate how they manifest in everyday primary care settings. Delayed identification of palliative care needs may result from limited knowledge, insufficient resources and reluctance to discuss sensitive topics, such as death, with patients and family members [30,31]. Previous approaches to facilitate early identification of palliative care needs include screening tools, but their performance is generally poor [32]. A United States study on palliative care in home healthcare emphasized the importance of a clearly designated clinician with responsibility for the patient to enable timely initiation of palliative care [33], and this is further supported by international qualitative evidence suggesting that the general practitioner, who have continuous relationships with patients, are well suited for a central role in palliative care provision [34]. This is an area with room for improvement in Sweden, where primary care has low patient-physician continuity compared to other European countries [35,36].

From a Scandinavian perspective, Norwegian qualitative studies indicate that general practitioners view their long-term patient relationships and broad clinical competence as important strengths when they work with palliative care [37,38]. However, the general practitioners role differs between Norway and Sweden (Box 1), particularly regarding practice organization and gatekeeping functions, which may limit transferability of findings [39,40]. Interestingly, urban Norwegian general practitioners described taking a more passive role in palliative care than those from rural settings in one qualitative study [37]. In our study, data was collected in urban Stockholm, which may have influenced experienced shortcomings in primary care involvement. This is supported by the Swedish palliative care registry data indicating high availability of specialised palliative care in Stockholm but fewer palliative consultations compared to other regions, suggesting a clearer divide between primary and specialised care [11].

Educational programs for primary care professionals have demonstrated positive effects on integrating palliative principles into primary care, but evidence that education alone reduces ED visits is limited [41,42]. Recent Swedish guidelines also highlight the importance of educational interventions [8], but further interventional research is needed to determine how these interventions should be implemented and whether they can increase the quality of home-based care and reduce avoidable emergency care use [42].

Despite the central role of primary care in supporting older adults with multiple long-term conditions near the end of life, the contribution of primary care professionals to treatment-limitation decisions and the initiation of palliative care remains insufficiently understood [43–45]. The Swedish national guidelines on palliative care emphasize initiation of palliative care early in the disease course, with integration across all care settings to ensure timely and person-centred support [8]. Strengthening the role of primary care in early recognition and management of palliative needs, ideally before crises arise, is crucial for reducing avoidable ED visits and enabling care in the patient’s preferred location. Achieving these goals will require adequate resources and sustained investment in primary care [8,41].

Findings from our study suggest that shared decision-making between healthcare professionals, patients, and families was insufficiently implemented. Engaging patients and their families in shared decision-making with discussions about goals of care and treatment limitations allows primary care teams to align care plans with patient preferences, potentially reducing ED visits and hospital admissions. However, both healthcare professionals and patients can hesitate to engage in conversations about treatment limitations and death [30,31]. Barriers for professionals include fear of negative reactions from patients or family members and time constraints, while patients may not anticipate or expect discussions about end-of-life decisions, or see the clear benefit of them [30,31]. This combination is likely to contribute to the lack of discussions on advanced care planning in primary care. Nevertheless, the demonstrated benefits of early palliative care suggest that these conversations, approached sensitively, remain essential [42]. This is particularly relevant when aiming to provide home-based palliative care, which most older adults prefer [9,10,46]. Expanding person-centred planning may reduce crisis-driven decisions and potentially avoidable ED visits, by ensuring that care aligns with patients’ goals and limiting unnecessary interventions and hospital admissions [46].

Our findings indicate that even when palliative care needs in older adults with multiple long-term conditions are recognized and preferences are established, delivery of palliative care can faulter through inadequate access to symptomatic treatment as well as gaps in communication and care coordination across health and social care providers. This may occur despite the high level of digitalization of health information systems in Sweden, where electronic health records are widely implemented [40]. However, persistent challenges in information transfer across care contexts – particularly between regional healthcare and municipal services such as home care and nursing homes – may limit the accessibility of key information in practice. These challenges can, in turn, contribute to ED visits. This underscores the importance of developing more integrated palliative care models and information systems spanning both healthcare and municipal care, to better support complex patients and prevent unnecessary ED use.

Historically, patients with cancer have had greater access to specialized palliative care than those with other life-limiting conditions [47], despite evidence that care needs are broadly similar across these conditions [48]. According to newly published Swedish guidelines, this imbalance persists: In 2024, nearly 80% of all patients who died with specialized palliative in Sweden care had cancer [8]. Yet active palliative care approaches have been shown to reduce ED use at the end of life and be cost-effective or cost-neutral compared to standard care with non-cancer conditions [47,49]. Despite this, much interventional research on primary care involvement in palliative care has focused on cancer patients [44]. This highlights the need for further interventional research on integrating palliative care into primary care for older adults with non-cancer life-limiting conditions. These patients are often cared for exclusively in primary care [8,11,20]. Ideally, primary care could provide high-quality palliative care for these patients, with support from specialist palliative care when needed [8,20,21,46]. However, to improve the quality of palliative care in primary care and reduce avoidable ED visits, it is essential to develop models that educate primary care professionals in identification and delivery of palliative care, and to address structural challenges, including information transfer and the provision of accessible care in both practices and the patient’s home.

## Methodological discussion

The data were originally collected to investigate avoidable ED visits among older adults with multiple long-term conditions. ED visits related to palliative care needs was interpreted as a prominent and analytically distinct theme that could not be explored in sufficient depth within the scope of the primary study, and was therefore analysed separately. As the original data collection was not specifically designed for this aim, some aspects of the phenomenon may not have been captured in the same depth that a dedicated palliative care study would have allowed. However, the fact that respondents elaborated on this issue without prompting suggests that it is an important and relevant area for further investigation.

Open-ended questions are frequently employed in qualitative and exploratory research [50]. Their main advantage lies in allowing participants to construct their responses freely, rather than selecting from predetermined options. This approach generates data that may not be accessible through theoretical reasoning alone and enables researchers to gain a more holistic and comprehensive understanding of the phenomenon under investigation [51].

Surveys are a resource-efficient method for collecting large volumes of data; however, they have inherent limitations, most notably, the inability to ask follow-up questions that might yield deeper insights into respondents’ reasoning [51]. We acknowledge that focus group discussions or individual interviews could have provided richer and more in-depth data through opportunities for probing and clarification. Nevertheless, the survey design enabled inclusion of respondents from multiple healthcare settings, capturing a broad range of experiences and perspective across providers. This made the dataset well suited for describing experiences of system-level shortcomings in healthcare delivery.

We consider this study to contribute to an important and underexplored area. However, the limitations of the survey design, together with the fact that the questions were not specifically designed to identify shortcomings in palliative care highlight the need for further qualitative and interventional research to deepen the understanding of the issues experienced and, importantly, how to achieve sustainable changes that support care aligned with patient preferences. Future research should also examine how barriers to high-quality palliative care in primary care settings can be addressed.

In the present study, the length of responses varied considerably, ranging from a few words or sentences to approximately half a page of text. Shorter responses occasionally made it difficult to discern what respondents perceived as the main causes of ED visits. Respondents frequently attributed potentially preventable ED visits to shortcomings in other parts of the healthcare system. Rather than indicating a lack of self-reflection, these responses likely reflect the fragmented nature of care for older adults and the challenges inherent in cross-organizational collaboration. Due to the survey design and non-iterative data collection, saturation or information power was not considered in the traditional qualitative sense. Instead, emphasis was placed on obtaining variation across professional groups and care contexts.

Although 44 of the 100 respondents mentioned palliative care-related issues in at least one free-text response, this should not be interpreted as a measure of the prevalence or relative importance of the phenomenon. Respondents were not explicitly asked about palliative care, and the extent to which they addressed the topic varied considerably. In qualitative research, the analytical significance of a theme is determined by the richness and explanatory value of the data rather than by the frequency with which it is mentioned. We therefore report the number of respondents for transparency while maintaining our focus on the content and meaning of the responses.

The study aimed to include healthcare professionals from multiple sectors along the care continuum to obtain a comprehensive understanding of potentially avoidable ED visits among older adults. However, it should be acknowledged that quota sampling does not ensure representativeness, as it relies on the researcher’s judgment when selecting participants [26]. Furthermore, the recruitment proved challenging, and it was therefore not possible to include the planned 20 respondents from each healthcare provider. As a result, some subgroups were small, with as few as ten respondents. This limitation should be considered when interpreting the findings. Nevertheless, the qualitative analysis, conducted across all respondents, provides a broad and multifaceted understanding, reflecting diverse professional perspectives. It is also noteworthy that the respondents of the original survey were highly experienced healthcare workers – nearly 70% reported more than ten years of professional experience – and that physicians, registered nurses, and nurse assistants were all represented.

Transferability of the results should be considered in relation to the study context and sample. Although the findings are not intended to be statistically generalisable, the experiences described may be relevant to similar healthcare contexts involving older adults with multiple long-term conditions. The recruitment procedure, in which managers contact persons distributed the survey, was intended to reach professionals with relevant experience and likely contributed to self-selection of engaged participants, a recognised feature of survey research that may enhance information richness in qualitative analyses. A potential limitation is that managers may have influenced participation by selecting individuals who align with their own views.

A further limitation is the uneven distribution of professional groups, with registered nurses and nurse assistants being the largest group of respondents. This may have influenced the findings by giving greater prominence to their perspectives, although all relevant professional groups were represented. At the same time, nurses typically have frequent and continuous contact with patients with palliative care needs across care settings, and may therefore have particular insight into factors contributing to ED visits. Another limitation of this separate analysis is that the free-text responses were anonymised before analysis, meaning that the 44 respondents included in this study could not be linked to individual respondent characteristics or compared with the full survey sample. This should be considered when interpreting the transferability of the results.

Finally, the perspectives of home care services and nursing home staff, as well as those of patients and family members, were not included in this study. Future research should incorporate these groups to achieve a more comprehensive understanding of the phenomenon.

## Conclusions

Healthcare professionals describe several shortcomings in outpatient settings that contribute to avoidable ED visits in older adults with multiple long-term conditions and palliative care needs. Late recognition of palliative needs emerges as a key and potentially modifiable driver. Strengthening primary care professionals’ palliative care competence through targeted education, alongside adequate organizational resources and access to specialist palliative support, is crucial. Better integration of generalist palliative care into primary care could reduce unnecessary ED visits while enabling more proactive, person-centred care aligned with patients’ end-of-life preferences.

## Data Availability

The data that support the findings of this study are not publicly available due to privacy and ethical restrictions but are available from the corresponding author upon reasonable request.
